# Supplementary material for: Causal Relationship between Aging and Anorexia Nervosa: A White-Matter-Microstructure-Mediated Mendelian Randomization Analysis
Source: Biomedicines. 2024 Aug 16;12(8):1874. doi: 10.3390/biomedicines12081874 (PMC11351342; doi:10.3390/biomedicines12081874)
Supplement: Supplementary file 1 [file biomedicines-12-01874-s001.zip › Supplementary Figure/Figure S4/Singe SNP plot of MR analysis for TL to ubm-b-1556.pdf]

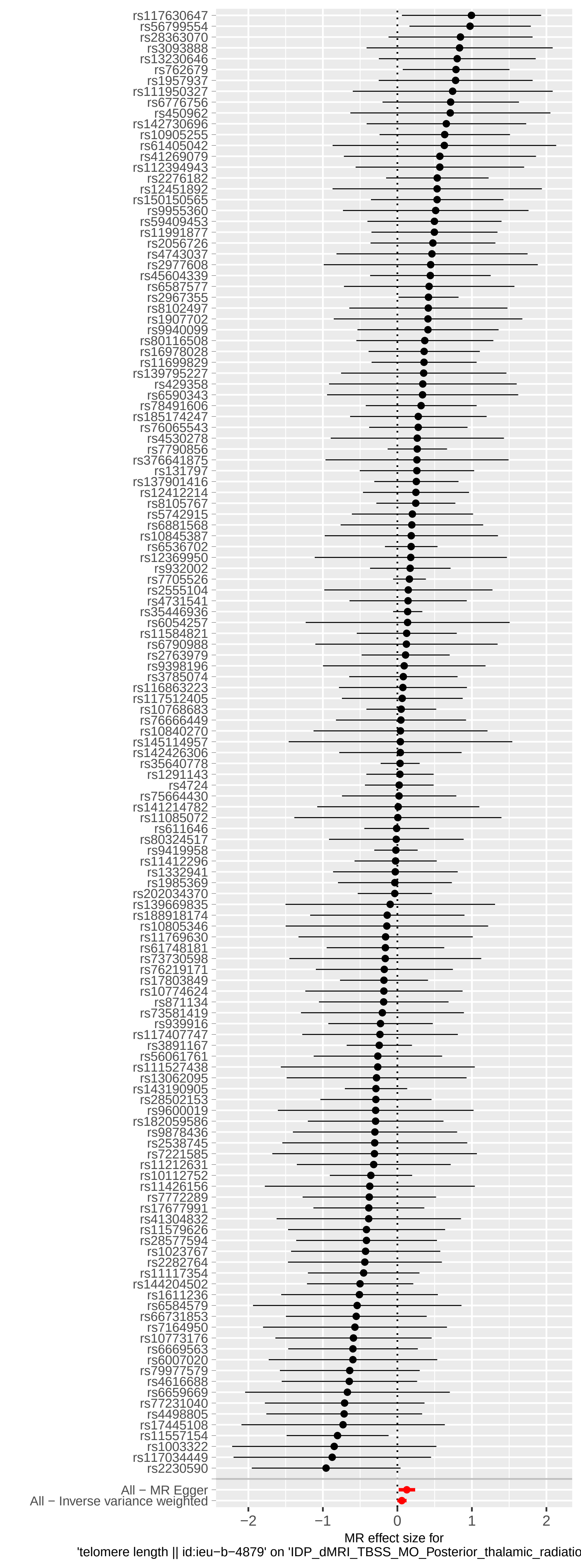
rs117630647
rs56799554
rs28363070
rs3093888
rs13230646
rs762679
rs1957937
rs111950327
rs6776756
rs450962
rs142730696
rs10905255
rs61405042
rs41269079
rs112394943
rs2276182
rs12451892
rs150150565
rs9955360
rs59409453
rs11991877
rs2056726
rs4743037
rs2977608
rs45604339
rs6587577
rs2967355
rs8102497
rs1907702
rs9940099
rs80116508
rs16978028
rs11699829
rs139795227
rs429358
rs6590343
rs78491606
rs185174247
rs76065543
rs4530278
rs7790856
rs376641875
rs131797
rs137901416
rs12412214
rs8105767
rs5742915
rs6881568
rs10845387
rs6536702
rs12369950
rs932002
rs7705526
rs2555104
rs4731541
rs35446936
rs6054257
rs11584821
rs6790988
rs2763979
rs9398196
rs3785074
rs116863223
rs117512405
rs10768683
rs76666449
rs10840270
rs145114957
rs142426306
rs35640778
rs1291143
rs4724
rs75664430
rs141214782
rs11085072
rs611646
rs80324517
rs9419958
rs11412296
rs1332941
rs1985369
rs202034370
rs139669835
rs188918174
rs10805346
rs11769630
rs61748181
rs73730598
rs76219171
rs17803849
rs10774624
rs871134
rs73581419
rs939916
rs117407747
rs3891167
rs56061761
rs111527438
rs13062095
rs143190905
rs28502153
rs9600019
rs182059586
rs9878436
rs2538745
rs7221585
rs11212631
rs10112752
rs11426156
rs7772289
rs17677991
rs41304832
rs11579626
rs28577594
rs1023767
rs2282764
rs11117354
rs144204502
rs1611236
rs6584579
rs66731853
rs7164950
rs10773176
rs6669563
rs6007020
rs79977579
rs4616688
rs6659669
rs77231040
rs4498805
rs17445108
rs11557154
rs1003322
rs117034449
rs2230590
All − MR Egger
All − Inverse variance weighted
−2
−1
0
1
2
MR effect size for
'telomere length || id:ieu−b−4879' on 'IDP_dMRI_TBSS_MO_Posterior_thalamic_radiatio
